# West Nile virus and arboviral threats: a call for integration into critical care preparedness

**DOI:** 10.1186/s44158-025-00276-5

**Published:** 2025-08-29

**Authors:** Giancarlo Ceccarelli, Gabriella d’Ettorre, Vlad Cristian Sanda, Marco Ridolfi, Francesco Alessandri

**Affiliations:** 1 Department of Public Health and Infectious Diseases, “Sapienza” University of Rome, Azienda Ospedaliero Universitaria Umberto I, Rome, Italy; 2 Department of General and Specialistic Surgery, Anesthesiology, “Sapienza” University of Rome, Azienda Ospedaliero Universitaria Umberto I, Rome, Italy

**Keywords:** West Nile virus, Critical infectious disease, Arboviral infections, Neuroinvasive disease

To the Editor,

The recent increase in West Nile virus (WNV) cases across Southern and Central Europe, including fatal neuroinvasive infections requiring intensive care unit (ICU) admission, underscores the urgent need to reconsider arboviral infections as emerging challenges for critical care in Europe. Despite being traditionally viewed as tropical or travel-associated, arboviruses—such as WNV, Dengue virus, Chikungunya virus, Usutu virus, Toscana virus—are now seasonally resurging in various European countries, driven by climate change, ecological shifts, and vector expansion [1, 2].

WNV in particular has shown an alarming trend. The 2022 and 2023 transmission seasons reported record numbers of human cases in Italy, Greece, Hungary, and Romania, including clusters of neuroinvasive disease with high morbidity and ICU occupancy [3]. As of August 7, 2025, Italy is experiencing an outbreak of West Nile virus (WNV) infection, with 173 confirmed human cases reported, including 72 presenting with neuroinvasive disease (WNND). Among these cases, 11 deaths have been notified, resulting in a case fatality rate of 15.2% for neuroinvasive forms—comparable to that observed in 2018, 2022, and 2024 (Fig. 1) [4].

**Fig. 1:**
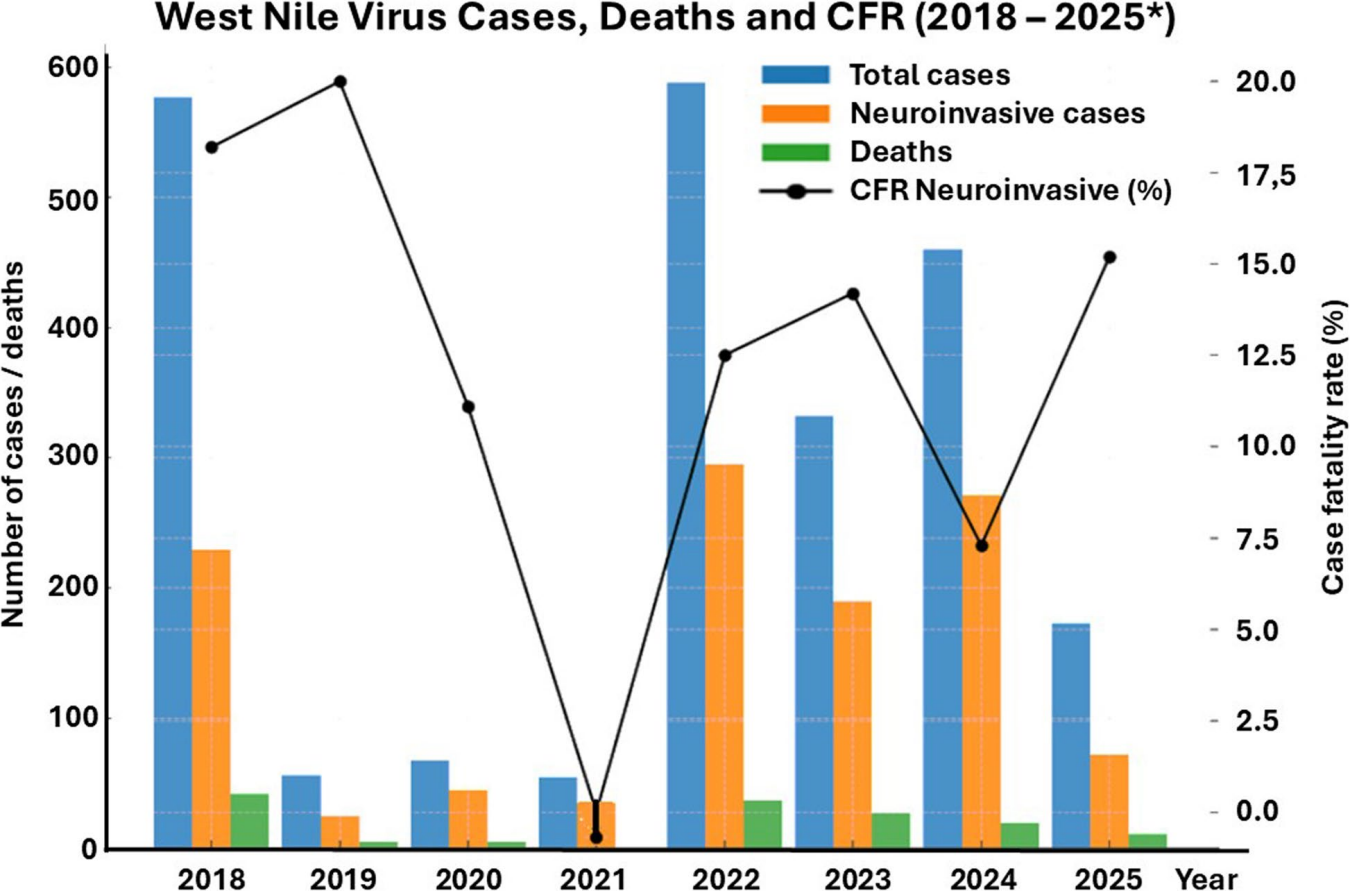
West Nile Virus (WNV) in Italy (2018–2025*): confirmed cases, neuroinvasive cases, reported deaths, and case fatality rate (CFR) in neuroinvasive cases. The bar plots display the total confirmed WNV cases, neuroinvasive cases, and reported deaths for each year. The line plot represents the fatality rate among neuroinvasive cases, shown as a percentage on the right *y*-axis. A notable fluctuation in both the incidence and severity (as reflected by CFR) is observed over time. *Data for 2025 are provisional and updated as of July 30, 2025

WNND can manifest as encephalitis, meningitis, or acute flaccid paralysis, and is frequently complicated by seizures, respiratory failure, and multiorgan dysfunction. These complications often necessitate prolonged ventilatory support and intensive monitoring, with case-fatality rates in ICU settings particularly high among older adults and immunocompromised patients [5, 6].

Despite these trends, awareness and preparedness among intensivists remain limited. Diagnostic suspicion is often delayed due to the non-specific presentation and lack of standardized ICU protocols for arboviral infections. Moreover, commercial diagnostic tools for WNV and related viruses are not routinely available in many hospitals, and testing often requires referral to national reference laboratories—introducing further delays in diagnosis and treatment [6].

There is also a growing concern for transfusion- and transplantation-related transmission in critically ill patients, especially during peak transmission months (July–October). While blood donor screening exists in some countries, ICU patients receiving multiple transfusions may remain at risk if such protocols are not uniformly applied [5–9].

We believe it is time for arboviral infections—especially WNV—to be formally integrated into ICU differential diagnoses during the summer and autumn months in Europe. This includes (Table 1):

- Incorporating arboviral panels into diagnostic workup for encephalitis and unexplained sepsis syndromes.
- Establishing ICU-specific protocols for supportive management of neuroinvasive disease.
- Enhancing cross-talk between infectious diseases, neurology, and critical care teams.
- Training clinicians on syndromic surveillance and early recognition of seasonal viral threats.
- Promoting real-time data sharing on arbovirus activity through ECDC and national public health authorities.

**Table 1:** Key clinical and public health priorities in response to the WNV epidemic

Priority area	Strategic action	Rationale	Implementation considerations
Diagnostic integration	Incorporate comprehensive arboviral panels (e.g., WNV, Usutu virus, Dengue) into the diagnostic workup for patients with encephalitis, acute flaccid paralysis, or unexplained sepsis.	Arboviruses are underdiagnosed in central nervous system (CNS) infections due to limited routine testing. Early identification improves patient management and public health response.	Ensure availability of multiplex PCR/serology in tertiary hospitals; update diagnostic algorithms; educate clinicians on test interpretation.
ICU protocol development	Establish ICU-specific protocols for supportive management of WNV neuroinvasive disease.	Neuroinvasive WNV can lead to severe complications such as cerebral edema, seizures, and respiratory failure. Structured protocols improve outcomes and resource allocation.	Develop protocols for neurological monitoring, intracranial pressure control, sedation strategies, and post-ICU rehabilitation; engage critical care societies.
Multidisciplinary coordination	Enhance collaboration between infectious disease specialists, neurologists, intensivists, and microbiologists through joint case reviews and interdepartmental coordination.	Complex neuroinfectious syndromes require integrated expertise for accurate diagnosis and effective management.	Establish regular interdisciplinary meetings, shared electronic health record (HER) notes, and cross-departmental communication pathways.
Clinical training and surveillance literacy	Train frontline clinicians on syndromic surveillance principles and recognition of seasonal arboviral threats.	Delays in clinical recognition contribute to late diagnosis and underreporting of arboviral cases. Surveillance literacy supports timely intervention.	Deliver continuing medical education (CME) modules, clinical decision support tools, and seasonal updates; prioritize training in emergency, neurology, infectious diseases, and primary care settings.
Surveillance and data sharing	Promote real-time data sharing on arbovirus circulation via European Centre for Disease Prevention and Control (ECDC) and national public health platforms.	Early warning systems and situational awareness are essential to prevent outbreaks and guide risk communication.	Develop regional arbovirus dashboards, standardize case definitions, and integrate entomological data; incentivize laboratory reporting.

Given the unpredictable nature of climate-driven outbreaks, the incorporation of arboviral preparedness into ICU systems is no longer optional—it is a necessary step toward resilient, seasonally adaptive intensive care.

## Supplementary Information

Supplementary Material 1.

## Data Availability

No datasets were generated or analysed during the current study.
