# Supplementary material for: West Nile virus and arboviral threats: a call for integration into critical care preparedness
Source: J Anesth Analg Crit Care. 2025 Aug 29;5:55. doi: 10.1186/s44158-025-00276-5 (PMC12395766; doi:10.1186/s44158-025-00276-5)
Supplement: Supplementary file 1 — Supplementary Material 1. [file 44158_2025_276_MOESM1_ESM.docx]

**Table 1S.** Key Clinical and Public Health Priorities in Response to the WNV Epidemic

| **Priority Area** | **Strategic Action** | **Rationale** | **Implementation Considerations** |
| --- | --- | --- | --- |
| Diagnostic Integration | Incorporate comprehensive arboviral panels (e.g., WNV, Usutu virus, Dengue) into the diagnostic workup for patients with encephalitis, acute flaccid paralysis, or unexplained sepsis. | Arboviruses are underdiagnosed in central nervous system (CNS) infections due to limited routine testing. Early identification improves patient management and public health response. | Ensure availability of multiplex PCR/serology in tertiary hospitals; update diagnostic algorithms; educate clinicians on test interpretation. |
| ICU Protocol Development | Establish ICU-specific protocols for supportive management of WNV neuroinvasive disease. | Neuroinvasive WNV can lead to severe complications such as cerebral edema, seizures, and respiratory failure. Structured protocols improve outcomes and resource allocation. | Develop protocols for neurological monitoring, intracranial pressure control, sedation strategies, and post-ICU rehabilitation; engage critical care societies. |
| Multidisciplinary Coordination | Enhance collaboration between infectious disease specialists, neurologists, intensivists, and microbiologists through joint case reviews and interdepartmental coordination. | Complex neuroinfectious syndromes require integrated expertise for accurate diagnosis and effective management. | Establish regular interdisciplinary meetings, shared electronic health record (HER) notes, and cross-departmental communication pathways. |
| Clinical Training and Surveillance Literacy | Train frontline clinicians on syndromic surveillance principles and recognition of seasonal arboviral threats. | Delays in clinical recognition contribute to late diagnosis and underreporting of arboviral cases. Surveillance literacy supports timely intervention. | Deliver continuing medical education (CME) modules, clinical decision support tools, and seasonal updates; prioritize training in emergency, neurology, infectious diseases, and primary care settings. |
| Surveillance and Data Sharing | Promote real-time data sharing on arbovirus circulation via European Centre for Disease Prevention and Control (ECDC) and national public health platforms. | Early warning systems and situational awareness are essential to prevent outbreaks and guide risk communication. | Develop regional arbovirus dashboards, standardize case definitions, and integrate entomological data; incentivize laboratory reporting. |
